# NiS submicron cubes with efficient electrocatalytic activity as the counter electrode of dye-sensitized solar cells

**DOI:** 10.1098/rsos.180186

**Published:** 2018-08-15

**Authors:** Qiongzhe Yu, Yashuai Pang, Qiwei Jiang

**Affiliations:** Henan Key Laboratory of Photovoltaic Materials, School of Physics and Electronics, Henan University, Kaifeng 475001, People's Republic of China

**Keywords:** NiS, counter electrode, dye-sensitized solar cells, electrocatalytic

## Abstract

In this work, nickel sulfide (NiS) submicron cubes, synthesized by an easy hydrothermal method, were investigated as an efficient electrocatalytic material of dye-sensitized solar cells (DSSCs), to our knowledge, for the first time. Part of the NiS submicron cubes were grown together in a hydrothermal procedure and formed the connected submicron cube cluster. The NiS submicron cubes (with a diameter of 300–800 nm) showed excellent electrocatalytic activity and presented superior photovoltaic performance when it was used as an electrocatalytic material for the counter electrode (CE) of DSSCs. The CE composed of the NiS submicron cubes could achieve a photovoltaic efficiency of 6.4%, showing their superior performance compared with the typical Pt electrode (which with the corresponding conversion efficiency was 5.3% at the same condition). The low-cost NiS submicron cube electrode could be a competitive candidate to replace the traditional Pt electrode in DSSCs. The simple composition procedure of NiS submicron cubes could enable the low-cost mass production of an efficient NiS submicron cube electrode to be easily accomplished.

## 1. Introduction

Dye-sensitized solar cells (DSSCs) have been one of the most attractive research hotspots in recent years because of the advantages of high conversion efficiency and low cost [1,2]. The DSSC is composed of a dye-sensitized semiconductor photo-anode, redox electrolyte and counter electrode (CE) [3,4]. As one of the most important parts of the DSSC, the CE is used to transmit electrons and promote the regeneration of I^−^ from I_3_^−^ [5–8]; this essential function needs the CE to possess excellent electron conductivity and outstanding electrocatalytic activity at the same time [4,9]. The CE of DSSCs is usually made of noble metal platinum (Pt) owing to its high electron conductivity, good stability and excellent electrocatalytic activity [10,11]. However, Pt is very expensive because of the scarce geological reserves [12,13], so it is highly important to develop new low-cost CE materials with a relatively high efficiency for DSSCs. Up to now, various materials, such as carbon-based materials [14–16], metal nitrides [10,17–19], metal oxides [20–22], metal sulfides [23,24], metal selenides [25–28], metal alloys [29–32], conducting polymers [4,28,33–36], metal carbides [37] and metal phosphides [38] have been extensively studied as cost-effective alternatives to replace the traditional Pt for DSSCs. Among these materials, the low-cost nickel sulfide (NiS) exhibits great potential as the CE of DSSCs owing to its high conductivity and excellent electrocatalytic activity. Over the last few years, NiS with various morphologies (nanorod, nano nanowall, nanosheets, etc.) has been synthesized using different chemical methods [24,37–39]. It was found that the morphology and the structure of the relevant material affect its catalytic properties greatly [40–47]. How to regulate and control the morphology of NiS is an important problem to be further researched. In this work, NiS submicron cubes, prepared by an easy hydrothermal method, were investigated as an efficient electrocatalytic material for the CE. To the best of the authors' knowledge, this is the first report on the synthesis of NiS submicron cubic grains and its potential applications for DSSCs.

## 2. Experimental sections

### 2.1. Preparation of NiS submicron cubes counter electrode

Amounts of 12.2 mmol NiSO_4_·6H_2_O (Aldrich Co., USA) and 16.5 mmol NaOH (Aladdin Co., China) were dissolved in 100 ml of distilled water, respectively, under vigorous stirring, and a green precipitate was formed in the mixture. The green precipitate was isolated by centrifugation and washed with absolute ethanol three times. Then the green precipitate and 0.6 g of sulfur (Aladdin Co., China) were added in a beaker containing 70 ml toluene (Aladdin Co., China) under strong stirring to form a mixture which was transferred into a 100 ml polytetrafluoroethene autoclave. The autoclave was heated at 160°C for 1 h and subsequently heated at 200°C for 5 h. Then after being freedom cooled to room temperature, the resulting black precipitate composed of NiS submicron cubes was washed with toluene and ethanol, respectively. The as-prepared NiS submicron cube was dispersed in ethanol, the NiS submicron cube electrode (about 10 µm in thickness) was prepared by coating a layer of NiS submicron cubes onto the surface of the fluorine-doped transparent conductive oxide (FTO) with the doctor scraping method. The NiS submicron cube-400 electrode (about 10 µm in thickness) was obtained through sintering the NiS submicron cube CE at 400°C for 1 h in the tube furnace with a flowing argon atmosphere.

### 2.2. Preparation of Pt counter electrode

The mirror-like Pt CE was obtained by electrodepositing a Pt layer on the surface of FTO (15Ω/sq, Nippon Sheet Glass Co., Japan) [48].

### 2.3. Fabrication of dye-sensitized solar cells

The dye-sensitized TiO_2_ electrode was prepared similarly to that reported in [49,50]. Titanium iso-propoxide (20 ml, 98+%, Fluka Co., USA) was added to the 0.1 M nitric acid (Aladdin Co., China) aqueous solution (120 ml) under violent stirring. After ageing at 80°C for 8 h, the solution became a translucent blue–white colloidal suspension. The resultant colloidal suspension was autoclaved at 240°C for 12 h to form a white slurry. The resultant slurry was concentrated and added to PEG-20000 (Aladdin Co., China) (20 wt% of the TiO_2_) to form a TiO_2_ colloid (12%). The TiO_2_ colloid was coated on the FTO glass using the doctor scraping method to form the TiO_2_ film. The TiO_2_ film was sintered at 450°C for 30 min in the electric furnace [51] and then was treated in a 40 mM TiCl_4_ aqueous solution at 70°C for 30 min, followed by subsequent sintering at 450°C in air for 30 min in the electric furnace. After the as-formed TiO_2_ electrode (with a TiO_2_ film thickness of 12 µm) was cooled to room temperature, it was immersed in a 2.5 × 10^−4^ M ethanol solution of Ruthenium 535 bis-TBA (N719, Solaronix SA) for 24 h to absorb the dye [52]. The electrolyte was composed of 0.3 M 1,2-dimethyl-3-propylimidazolium iodide (DMPII), 0.03 M I_2_, 0.5 M 4-tert-butyl pyridine and 0.1 M guanidinium thiocyanate, and the solvent of the electrolyte was acetonitrile [53]. The electrolyte was injected into the interspace between the dye-sensitized TiO_2_ electrode and the CE to form a solar cell. The cell was sealed with solid paraffin to prevent the leakage of electrolyte. The effective cell area was 0.25 cm^2^.

### 2.4. Materials and dye-sensitized solar cell characterizations

The structure and morphology of the resultant samples were detected using X-ray diffraction (XRD, Rigaku D/max-2500), scanning electron microscopy (SEM, Hitachi S-3500N) and transmission electron microscopy (TEM, FEI Tecnai 20). The DSSCs were illuminated by a solar simulator (CHF-XM500, Beijing Trusttech Co. Ltd) under 100 mW cm^−2^ (calibrated by a standard silicon solar cell) irradiation. The photocurrent–voltage (*I–V*) characteristic curves of the DSSCs under simulated sunlight were recorded using an RST5200D (Shiruisi, China) electrochemical workstation. Both the Tafel curve and the electrochemical impedance spectra (EIS) of the counter electrodes were measured using the same electrochemical workstation. The EIS in the symmetric cell configuration with two identical counter electrodes were measured with an AC modulation signal of 20 mV and a bias voltage of 0.3 V, with the frequency range from 100 kHz to 100 MHz.

## 3. Results and discussion

**Figure 1.**
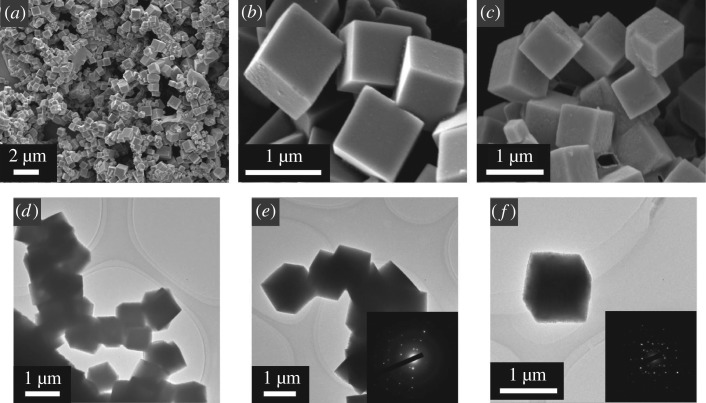
SEM images of NiS submicron cubes (*a*,*b*) and NiS submicron cubes-400 (*c*). TEM images of NiS submicron cubes (*d*,*e*) and NiS submicron cubes-400 (*f*).

Figure 1 shows the SEM and TEM images of the as-prepared NiS submicron cubes which exhibit cubic shape and possess smooth surfaces. For the NiS submicron cubes, most of the diameters are about 300–800 nm; part of the NiS submicron cubes were grown together by the hydrothermal *in situ* growth procedure and formed the cubic grains cluster. The NiS submicron cubic grains could keep the cube shape after it was sintered for 1 h at 400°C in the argon atmosphere; only the surface of the cubes became relatively rough (figure 1*c*,*f*). The selected area electron diffraction (SAED) (figure 1*e*) clearly shows the single crystal diffraction pattern of the hexagonal NiS; this means that each of the NiS submicron cubic grains is composed of a single crystal. We found that along with the hydrothermal time increases, the diameter of the NiS submicron cubes could gradually increase, and the growth mechanism of the grains is likely to be the hydrothermal growth of NiS submicron cubes accompanied by the dissolution of smaller NiS grains.

**Figure 2.**
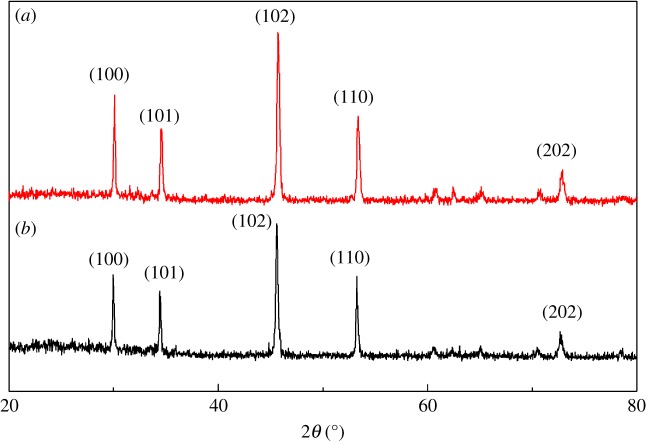
XRD patterns of NiS submicron cubes (*a*) and NiS submicron cubes-400 (*b*).

Figure 2*a* gives the XRD patterns of the NiS submicron cubes; all the diffraction peaks can be indexed to hexagonal NiS (JCPDS No.02-1273). After the NiS submicron cubes were sintered for 1 h at 400°C in the argon atmosphere, the pure hexagonal phase remained according to the XRD pattern, as exhibited in figure 2*b*. All the SAED patterns of the samples in the TEM images are consistent with the parameters of the XRD patterns shown in figure 2. The main difference between the diffractions in figure 2 is that the intensity of the peaks were reduced after the NiS submicron cubes were sintered for 1 h at 400°C in the argon atmosphere. This is probably because the high temperature could lead to some potential signs of phase transition in the crystal structure, which leads to a small decrease in the crystallinity of the NiS powder.

**Figure 3.**
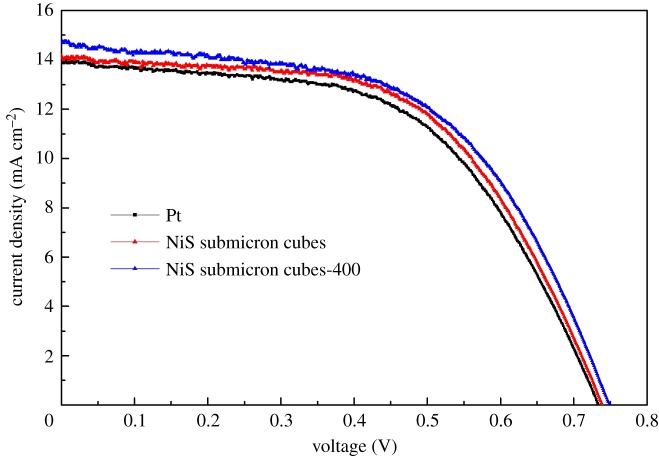
*I*–*V* characteristic curves of DSSCs with different counter electrodes of Pt, NiS submicron cubes and NiS submicron cubes-400, which were measured under simulated sunlight (100 mW cm^−2^, AM 1.5).

Figure 3 shows the *I–V* characteristics curves of the DSSCs based on the CE of Pt, NiS submicron cubes and NiS submicron cubes-400, respectively. The detailed photovoltaic parameters from the *I–V* curves are summarized in table 1. As a reference, when using a typical Pt electrode as the CE, the corresponding DSSC has a power conversion efficiency (*η*) of 5.3%. When using the NiS submicron cube electrode as the CE, the corresponding *η* is 5.9%, showing its superior performance compared with the Pt electrode. When using the NiS submicron cube-400 electrode as the CE, the corresponding *η* was further increased to 6.4%, demonstrating the positive effect of the calcination process at 400°C in an argon atmosphere.

**Table 1.** The photovoltaic parameters of DSSCs with different CEs.

CE	*J*_SC_ (mA cm^−2^)	*V*_OC_ (V)	FF	*R*_S_ (Ω)	*R*_CT_ (Ω)	*η* (%)
Pt	14.1	0.735	0.51	7.41	7.37	5.3
NiS submicron cubes	14.2	0.741	0.56	6.95	5.06	5.9
NiS submicron cubes-400	14.7	0.748	0.58	6.43	2.99	6.4

**Figure 4.**
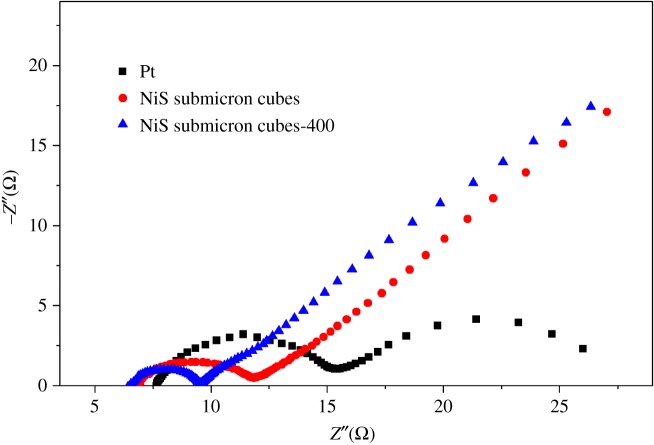
Nyquist plots of the symmetric cells with two identical counter electrodes of Pt, NiS submicron cubes and NiS submicron cubes-400.

To further evaluate the electrochemical feature of the as-prepared NiS electrode, the EIS were measured in the symmetric (CE–CE) cell. The EIS of different electrodes are shown in figure 4 and the parameters from the EIS are summarized in table 1. The *R*_CT_ (surface charge-transfer resistance) of the Pt electrode is 7.41 Ω, indicating a good electrocatalytic activity. The *R*_CT_ of the NiS submicron cubes electrode is 5.06 Ω, which means that the NiS submicron cube electrode provides a much better electrocatalytic activity. In the case of the NiS submicron cube-400 electrode, the *R*_CT_ was further reduced to 2.99 Ω, much smaller than the that of the typical Pt electrode (5.63 Ω), which results in an excellent conversion efficiency. This is probably because after the NiS submicron cubes were sintered for 1 h at 400°C in the argon atmosphere, the connections at the interfaces of the submicron cubes became closer, which could be seen from the sheet resistance (*R*_S_). The *R*_S_ of the NiS submicron cube electrode is 6.95 Ω; the *R*_S_ of the NiS submicron cube-400 electrode was decreased to 6.43 Ω, showing better conductivity. The *R*_S_ of the NiS submicron cube electrode based on FTO is a little greater compared with that of the large block of NiS or the metal-based CE [7,11], which also means that there is still scope to further improve the conductivity of the NiS electrode and correspondingly to improve its photovoltaic efficiency. The NiS submicron cube electrode is important in that it offers a new low-cost CE with novel cubic morphology.

**Figure 5.**
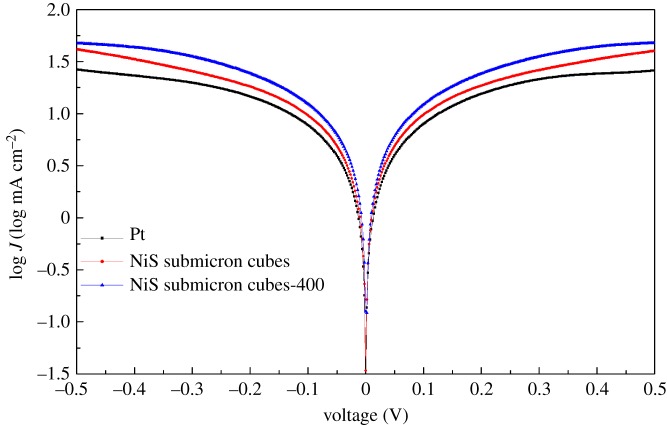
Tafel curves of the symmetric cells with two identical counter electrodes of Pt, NiS submicron cubes and NiS submicron cubes-400.

To further investigate the catalytic activities of the CEs, the Tafel curves were measured in the symmetric cell (CE–CE cell) configuration which consists of two identical CEs. Figure 5 shows the Tafel curves of the Pt, NiS submicron cubes and NiS submicron cubes-400 electrodes. The limiting current density (*J*_lim_) and the exchange current density (*J*_0_) are related to the catalytic activity of the catalysts. The Tafel curve based on the NiS submicron cube electrode shows a relative higher *J*_0_ and *J*_lim_ compared with that of the Pt electrode. The higher *J*_0_ and *J*_lim_ mean that the NiS submicron cube electrode has better catalytic activity for the I_3_^−^ reduction. The relative trend of these parameters indicates that the catalytic performance of the NiS electrode is superior to that of the Pt electrode, which is similar to that reported in the literature [12,30]. It is noted that after the calcination process, the as-formed NiS submicron cube-400 electrode shows a further improved *J*_0_ and *J*_lim_ compared with the NiS submicron cube electrode, showing the best catalytic property among the three CEs.

## 4. Conclusion

In summary, the NiS submicron cubic grains with a uniform cube shape, prepared by the hydrothermal method, were investigated as the CE material of DSSCs, to the best of our knowledge, for the first time. It was found that the electrodes composed of NiS submicron cubes show excellent catalytic activity and high photoelectric conversion efficiency competing with a typical Pt CE. It is noted that the simple composition procedure of the NiS submicron cubes could enable the low-cost mass production of the NiS submicron cube electrode to be easily accomplished. The low-cost NiS material with a uniform submicron cube shape and excellent catalytic activity could be a potential alternative to the traditional expensive noble metal Pt for DSSCs.

## Supplementary Material

Equivalent circuit and the EDS spectrum

## Data accessibility

All of the data in this study are included in figures and the electronic supplementary material, and freely available.
