# Supplementary material for: NiS submicron cubes with efficient electrocatalytic activity as the counter electrode of dye-sensitized solar cells
Source: R Soc Open Sci. 2018 Aug 15;5(8):180186. doi: 10.1098/rsos.180186 (PMC6124119; doi:10.1098/rsos.180186)
Supplement: Equivalent circuit and the EDS spectrum [file rsos180186supp1.pdf]

ROYAL SOCIETY
OPEN SCIENCE

R. Soc. open sci.
doi:10.1098/not yet assigned

Electronic supplementary Material

NiS submicron cubes with efficient electrocatalytic activity as the counter electrode of dye-sensitized solar cells

Qiongzhe Yu, Yashuai Pang and Qiwei Jiang*

Henan Key Laboratory of Photovoltaic Materials, School of Physics and Electronic, Henan University, Kaifeng 475001, China.

Keywords: NiS, counter electrode, dye-sensitized solar cells, electrocatalytic

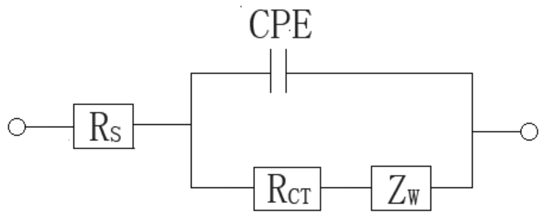

Fig. S1 Equivalent circuit of EIS for the CE-CE cell. R_s is a sheet resistance, CPE is a constant phase element, R_{CT} is a charge-transfer resistance, Z_W is Nernst diffusion impedance.

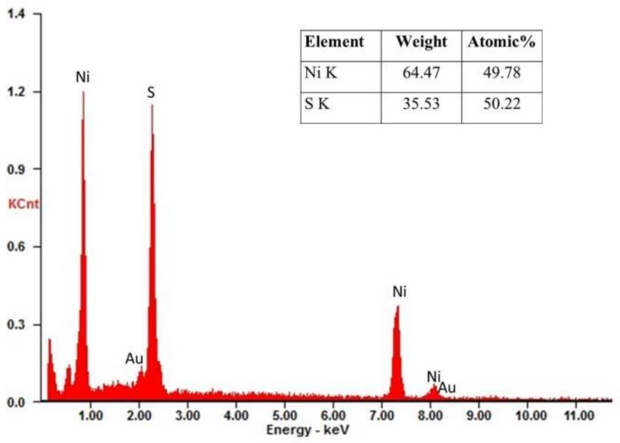

Fig. S2 The EDS spectrum of the as prepared NiS submicron cubes
